# Game-theoretic analysis of governance and corruption in China's pharmaceutical industry

**DOI:** 10.3389/fmed.2024.1439864

**Published:** 2024-08-14

**Authors:** Xi Wang, Tao Zhang, Hanxiang Gong, Jinghua Li, Baoling Wu, Baoxin Chen, Shufang Zhao

**Affiliations:** ^1^Faculty of Humanities and Social Sciences, Macau Polytechnic University, Macau, Macau SAR, China; ^2^The Second Affiliated Hospital, Guangzhou Medical University, Guangzhou, Guangdong, China; ^3^School of Public Health, Guangzhou Medical University, Guangzhou, Guangdong, China; ^4^Pingshan Hospital, Southern Medical University, Shenzhen, Guangdong, China

**Keywords:** evolutionary game theory, governance, corruption, pharmaceutical industry, regulatory strategies, public health safety

## Abstract

**Introduction:**

With the rapid development of China's pharmaceutical industry, issues of corruption and regulatory effectiveness have become increasingly prominent, posing critical challenges to public health safety and the industry's sustainable development.

**Methods:**

This paper adopts a bounded rationality perspective and employs a game-theoretic evolutionary approach to establish a tripartite evolutionary game model involving pharmaceutical companies, third-party auditing organizations, and health insurance regulatory agencies. It analyzes the stable strategies of the parties involved and the sensitivity of key parameters within this tripartite game system.

**Results:**

The study reveals that adherence to health insurance regulations by pharmaceutical companies, refusal of bribes by third-party auditing organizations, and the implementation of lenient regulations by health insurance agencies can form an effective governance equilibrium. This equilibrium state contributes to reducing corruption in the pharmaceutical industry, balancing the interests of all parties, and promoting healthy industry development.

**Discussion:**

Pharmaceutical companies must balance compliance costs against the risks of non-compliance benefits while maximizing profits; third-party auditing organizations need to choose between fulfilling their duties and accepting bribes, considering their economic benefits and professional reputation; health insurance regulatory agencies adjust their strategies between strict and lenient regulation to maximize social welfare. The paper suggests enhancing policy support, strengthening compliance supervision, improving audit independence, and adjusting regulatory strategies to optimize governance in the pharmaceutical industry. Additionally, the research highlights the role of collaborative efforts among the three parties in achieving sustainable governance. Furthermore, the study conducts a numerical simulation analysis to demonstrate the impact of various parameters on the evolutionary stability of the system, providing practical insights into the implementation of regulatory policies. This research offers new insights for policy formulation and governance in China's pharmaceutical sector, providing significant reference value for guiding the industry's sustainable development.

## 1 Introduction

As China's economy continues to grow, the pharmaceutical industry has become a vital component of the national economy. However, the Chinese pharmaceutical sector has long been plagued by significant corruption issues, which not only undermine the efficacy and quality of medical services but also severely impact the healthy development of the industry and public trust in the entire medical system (1, 2). Corruption in the pharmaceutical industry includes, but is not limited to, bribery, illegal kickbacks, and the abuse of public office. These practices not only contravene legal provisions but also disrupt the fair allocation of medical resources. Similar challenges are faced globally by the pharmaceutical industry. For example, some major pharmaceutical companies in the United States have been fined heavily for illegal marketing practices, while incidents of bribery and fraud in medical procurement are common in some countries in Africa and South America (3–6). These corruption issues reflect the widespread nature of pharmaceutical industry corruption globally and the complex challenges it poses. Corrupt practices hinder medical innovation, equitable access to medical resources, increase the burden on public health systems, and diminish the effectiveness of treatments and patient satisfaction. For example, the bribery scandal involving GlaxoSmithKline in China in 2013 significantly impacted the company's ability to innovate and maintain market trust (3). Furthermore, a study by Mackey and Cuomo highlights how corruption in drug procurement processes in low- and middle-income countries leads to inequitable access to essential medicines, thereby increasing the public health burden (4).

China places high importance on combating commercial bribery in the pharmaceutical industry, enacting a series of laws and policy measures aimed at regulating the industry's order and promoting healthy development. For instance, the Anti-Unfair Competition Law of the People's Republic of China, enacted in 2018, explicitly prohibits commercial bribery and other unfair competition practices, with Article 8 specifically addressing the prohibition of commercial bribery, including any form of bribe given to a counterparty's staff for the purpose of securing business transactions (PRC Anti-Unfair Competition Law, 2018)^1^. The Drug Administration Law of the People's Republic of China, promulgated in 2019, enhanced drug approval and market supervision, with Article 39 requiring comprehensive documentation and transparency throughout the drug production, distribution, and usage processes, and Article 75 imposing stricter penalties for legal violations (PRC Drug Administration Law, 2019). The Anti-Monopoly Law, revised in 2022, strengthened regulation against potential monopolistic behaviors in the pharmaceutical industry, particularly through Article 17 which addresses the abuse of market dominance, promoting fair market competition (PRC Anti-Monopoly Law, 2022)^2^. The implementation of these laws and regulations provides legal and institutional support for corruption prevention and rectification in the pharmaceutical field, representing key measures to drive the continuous and healthy development of the industry.

However, relying solely on the formulation of laws and policies is insufficient; effective supervision and anti-corruption mechanisms are equally crucial. In the pharmaceutical industry, the complexity of supervisory entities, the diversity of regulatory tools, and the covert nature of the entities being regulated make supervision challenging. Therefore, understanding the interplay between policy, supervision, and governance is vital for enhancing regulatory standards in the pharmaceutical industry and effectively addressing corruption issues.

This study focuses on the three main stakeholders in China's pharmaceutical industry—pharmaceutical companies, third-party auditing organizations, and health insurance regulatory agencies—as the core of the study. By analyzing the roles, interrelationships, and strategic interactions of these three parties in addressing corruption, the paper reveals their mechanisms of action and influencing factors in corruption within the pharmaceutical industry. Furthermore, the paper explores how improving the coordination and cooperation among these three parties and strengthening regulatory mechanisms can effectively tackle corruption in the pharmaceutical industry, promote its healthy development, and safeguard public interest. To achieve these objectives, this study adopts a bounded rationality perspective and employs a game-theoretic evolutionary approach. We establish a tripartite evolutionary game model involving pharmaceutical companies, third-party auditing organizations, and health insurance regulatory agencies. The model analyzes the stable strategies of the parties involved and the sensitivity of key parameters within this tripartite game system.

In summary, this study aims to address the persistent and pervasive corruption issues in China's pharmaceutical industry, which threaten public health and the industry's sustainable development. By employing a game-theoretic evolutionary approach, we seek to explore the strategic interactions among key stakeholders—pharmaceutical companies, third-party auditing organizations, and health insurance regulatory agencies—and identify stable anti-corruption strategies that can effectively mitigate corruption and promote industry growth. Through an in-depth analysis of the strategic interactions among pharmaceutical companies, third-party auditing organizations, and health insurance regulatory agencies, this paper aims to provide theoretical insights and policy recommendations for resolving corruption issues in the pharmaceutical sector.

## 2 Literature review

### 2.1 Corruption in the pharmaceutical industry: historical context and modern challenges

Corruption in the pharmaceutical industry exhibits diversity and persistence, with a complex historical background. From the early days of unregulated competition in the drug market to the regulatory challenges of the modern globalized context, the industry has undergone significant transformations. Initially, the lack of effective regulatory mechanisms led to the proliferation of substandard and counterfeit drugs (7). Since the mid-20th century, as drug approval and market supervision systems were gradually established, the forms of corruption in the pharmaceutical industry became increasingly complex, involving drug price manipulation, falsification of clinical trial data, and inappropriate marketing practices (8). These practices not only endanger public health but also undermine the ethical standards and public trust in the pharmaceutical industry.

Currently, the pharmaceutical industry faces a more diverse and complex set of corruption challenges. Globalization has introduced problems with transnational drug supply chains, the highly competitive market environment, and increasingly complex drug approval processes, all of which provide fertile ground for corrupt practices (9–11). For example, some pharmaceutical companies exploit their market dominance to manipulate prices, or engage in opaque collaborations with doctors and medical institutions, influencing the promotion and use of drugs (3, 5). Additionally, the falsification of clinical trial data has become increasingly scrutinized in recent years, affecting the safety and efficacy of drugs and damaging the scientific credibility of the entire industry (8). Therefore, strengthening regulations, enhancing transparency, and intensifying the punishment of corrupt practices are key challenges currently faced by the pharmaceutical industry.

### 2.2 Governance of corruption issues in China's pharmaceutical industry

The causes of corruption in the pharmaceutical sector are complex, intertwining historical and contemporary factors, and exhibit both complexity and specificity. Corruption in this field spans various segments including drug distribution, medical examinations and treatments, equipment procurement, supply of consumables, and construction projects. It involves a complex array of personnel and a variety of corrupt practices, making investigations particularly challenging; thus, anti-corruption efforts in healthcare represent a prolonged battle (12, 13). For instance, the Changsheng Biotechnology vaccine scandal in 2018 revealed severe violations in the production of rabies vaccines, including the falsification of production test records and product release data, providing false information during regulatory inspections, and bribing relevant department officials (14, 15). In 2023, Xu Qingfeng, the director of the Guangdong Provincial Administration of Traditional Chinese Medicine, was arrested for accepting substantial bribes. During his tenure, he exploited his position to aid several pharmaceutical companies in matters of drug procurement and project approval, illegally profiting from these transactions (16).

The root causes of corruption in China's pharmaceutical industry primarily stem from an imperfect regulatory system, fierce market competition, and a cultural reliance on networking. These factors collectively create a fertile ground for corruption (17, 18): Regulatory bodies, lacking sufficient human and technical resources, struggle to effectively supervise the broad and complex pharmaceutical industry. The uneven enforcement of laws, combined with local protectionism, further weakens the deterrent power of regulations. Moreover, the high profits at stake drive companies to resort to illegal methods to gain competitive advantages in the fierce market (17, 18). Additionally, the deeply ingrained culture of relationships provides a covert social acceptance for such behaviors, contributing to the longstanding and complex nature of corruption within the industry (17).

In recent years, the pharmaceutical industry in China has made significant progress in anti-corruption efforts, reflecting the government's strong commitment to combating corruption. The government has not only revised laws and regulations such as the Drug Administration Law and the Anti-Unfair Competition Law, thereby strengthening the penalties for illegal activities, but it has also enhanced the regulatory system by increasing industry transparency and promoting public participation, systematically addressing and preventing corrupt practices. Additionally, the use of digital and information technology tools, such as electronic tracking systems and online monitoring platforms, has enhanced the government's ability to monitor the distribution of drugs and medical services in real time. This has increased the likelihood of detecting corrupt activities and raised the cost of non-compliance (19, 20). Furthermore, the rise of social media and online complaint platforms has strengthened public engagement, promoting policy transparency and public oversight of medical practices (21). However, despite notable advancements in anti-corruption within the pharmaceutical sector, challenges remain in ensuring comprehensive transparency and eradicating deep-seated corruption (22). Anti-corruption efforts in the pharmaceutical industry continue to be a crucial focus of the Chinese government's broader campaign against corruption (23).

### 2.3 Game theory in pharmaceutical research: theory and practice

Game theory, a mathematical framework for analyzing strategic interactions, plays a crucial role in the application within the pharmaceutical industry (24). Historically, the multifaceted stakeholder relationships in the pharmaceutical sector have constituted complex game scenarios, including pharmaceutical companies, regulatory bodies, healthcare providers, and consumers. While each participant pursues their respective interests, their decisions are influenced by the choices of others (25–27). The essence of game theory lies in analyzing the strategic choices and outcomes under these interactions. For example, the decisions of pharmaceutical companies regarding research and development and market strategies depend not only on market demand and competitor behavior but are also constrained by policies, regulations, and public opinion. Concepts such as Nash equilibrium are employed to explain the strategic choices of all parties in a stable state (28–30).

In practice, game theory is extensively used to guide policy-making and managerial decisions in the pharmaceutical industry. For instance, when government agencies formulate regulatory policies for the pharmaceutical sector, they consider the market behaviors and potential reactions of pharmaceutical companies, aiming to achieve optimal social welfare (31, 32). Similarly, pharmaceutical companies facing intense market competition and increasingly stringent regulatory demands use game theory to optimize their business strategies, ensuring sustainable development and compliance (33). The integration of theory and practice not only deepens our understanding of the dynamics within the pharmaceutical industry but also provides a scientific basis for formulating more effective industry regulations (34). This interdisciplinary approach increasingly demonstrates its importance and efficacy in addressing complex issues within the pharmaceutical industry (35).

To enhance the understanding of these dynamics, it is essential to consider the broader applications of game theory in related fields. For example, Hua et al. (36) conducted a game-theoretic analysis of pricing and cooperative advertising in a reverse supply chain for unwanted medications in households, which highlights the strategic interactions and potential cooperative strategies that can be applied to the pharmaceutical industry. Li and Ma (37) explored financial reforms and regional investment conflicts in China through a game-theoretic lens, providing insights into how regulatory changes can influence stakeholder behavior in the pharmaceutical sector (37). Additionally, Tat et al. (38) developed a mathematical model for pharmaceutical supply chain coordination, emphasizing the importance of reselling medicines in an alternative market, which can inform strategies to combat corruption and inefficiencies in the pharmaceutical supply chain. Wen and Zhou (39) examined the impacts of regional governmental incentives on the straw power industry in China, using game theory to understand the implications of policy incentives on industry practices, which can be analogously applied to understand the effects of regulatory incentives in the pharmaceutical industry. Finally, Zhu (40) analyzed the deterrent effects of severe penalties on corruption through a game-theoretic approach, providing valuable insights into the effectiveness of stringent anti-corruption measures in the pharmaceutical industry.

### 2.4 The role and challenges of third-party auditing organizations in the pharmaceutical industry

In the pharmaceutical industry, Third-Party Auditing Organizations play a pivotal role. These organizations are primarily responsible for conducting independent reviews of the financial statements and business operations of pharmaceutical companies to ensure compliance with relevant laws, regulations, and industry standards. Third-party audits are indispensable for protecting public interests and safeguarding the rights of investors and consumers. Through these audit activities, financial irregularities, governance deficiencies, and potential unethical behaviors within the pharmaceutical industry can be uncovered, assisting regulatory bodies in timely identifying and addressing industry issues (41).

However, Third-Party Auditing Organizations face numerous challenges in fulfilling their duties. Firstly, the complexity of the pharmaceutical industry makes the auditing process more technical and specialized. The intricate financial structures and business processes of pharmaceutical companies demand auditors not only have profound expertise but also require adaptability (42, 43). Secondly, conflicts of interest pose a significant challenge. For instance, some auditing organizations may struggle to maintain complete independence due to economic ties, which can lead to questions about the reliability of audit results. With the rapid transformations in the pharmaceutical sector, including new regulatory policies and technological advancements, auditing agencies are tasked with the critical responsibility of adapting to these changes and effectively carrying out their auditing roles (44, 45).

## 3 Evolutionary model assumptions and description

### 3.1 Model description

In this study, we employ an evolutionary game theory model to analyze the interactions and behavioral patterns of pharmaceutical companies, Third-Party Auditing Organizations, and Medical Insurance Regulatory Agency in China's pharmaceutical industry, particularly in addressing corruption issues. The model focuses on the strategic choices of each party under economic incentives and punitive mechanisms, as well as their impact on the safety of health insurance funds and industry governance. Specifically, the model quantifies the decision-making processes of each entity through a set of variables: pharmaceutical companies decide between compliance and non-compliance with health insurance regulations, weighing the benefits and costs associated with each state; Third-Party Auditing Organizations choose between performing their duties and accepting bribes, balancing the costs of audits against potential rewards or penalties; and Medical Insurance Regulatory Agency focus on formulating and enforcing regulatory policies to maximize social welfare and minimize losses to the health insurance fund. These variables interact to create a dynamic game environment where parties adjust their strategies in response to others' actions and changes in the external environment. Through this model, the study aims to reveal the equilibrium states under different strategic combinations and their potential impacts on governance in the pharmaceutical industry, providing theoretical support for the formulation of effective policies and countermeasures. All variables used in the model and their meanings are listed in Table 1.

**Table 1:** Variables and variable description.

**Game participant**	**Variable code**	**Variable description**
Medical Insurance Regulatory Agency	*C*_1_	Cost incurred by the regulatory department in supervising pharmaceutical companies and third-party auditing organizations
	*L*_1_	Losses to the health insurance fund due to pharmaceutical companies not complying with regulations
	*L*_2_	Losses to the health insurance fund due to misconduct by third-party auditing organizations
	*N*_1_	Recovered amount from pharmaceutical companies: The amount of funds successfully recuperated by the government from non-compliant pharmaceutical companies
	*N*_2_	Recovered amount from third-party auditing organizations: The amount of funds successfully recuperated by the government from non-compliant third-party auditing organizations
	*F*_*h*_	Fines imposed on pharmaceutical companies: Penalties levied on non-compliant pharmaceutical companies
Pharmaceutical Companies	*W*_1_	Normal income under compliance: Normal business revenue of pharmaceutical companies when compliant
	*W*_2_	Income under non-compliance: Revenue of pharmaceutical companies when in violation of regulations
	*C*_2_	Sales cost: Cost of sales for pharmaceutical companies
	*R*_1_	Health insurance compensation under compliance: Compensation received by pharmaceutical companies under rule compliance
	*R*_2_	Health insurance benefits under non-compliance: Benefits gained by pharmaceutical companies from non-compliant collaborations with third parties
	*C*_3_	Speculative cost: Cost incurred by pharmaceutical companies in pursuing higher returns through high-risk, non-compliant strategies
Third-Party Auditing Organizations	*E*_*t*_	Cost of routine inspections carried out by third-party auditing organizations on pharmaceutical enterprises
	*S*_*t*_	Financial subsidies/awards provided by the health insurance department to third-party auditing organizations
	*F*_*s*_	Fines imposed by the Medical Insurance Regulatory Agency on third-party auditing organizations for violations
	*E*_*s*_	Losses/risks faced by third-party auditing organizations due to non-compliant operations
	*W*_3_	Normal earnings of third-party auditing organizations
	M	Additional earnings obtained by third-party auditing organizations from bribes received from pharmaceutical companies

**Figure 1:**
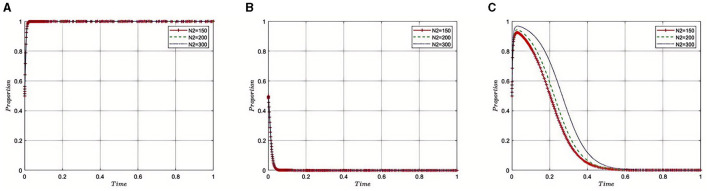
**(A–C)** Logogram of the evolutionary game between pharmaceutical companies, third-party auditors and health insurance regulators.

In the context of addressing governance and corruption within China's pharmaceutical industry, a complex tripartite game relationship forms between pharmaceutical companies, third-party auditing organizations, and health insurance regulatory agencies. Each party pursues its own interests and goals while being influenced by the actions of the other two, creating a dynamic decision-making environment. Within this evolutionary game framework, the interactions and feedback among pharmaceutical companies, third-party auditing organizations, and health insurance regulatory agencies collectively drive the governance process in the pharmaceutical sector. The logical relationships in this tripartite evolutionary game are illustrated in Figure 1.

### 3.2 Evolutionary model assumptions

This study employs the framework of evolutionary game theory to analyze the strategic interactions among pharmaceutical companies, Third-Party Auditing Organizations, and Medical Insurance Regulatory Agency in the Chinese pharmaceutical sector, and their impact on industry governance. Based on this, we propose the following assumptions to construct and analyze the corresponding evolutionary model:

Assumption 1: Key Participants. In the context of corruption in the Chinese pharmaceutical industry, the primary participants include pharmaceutical companies, Third-Party Auditing Organizations, and Medical Insurance Regulatory Agency. This study constructs an evolutionary game model involving these three parties, assuming that each makes strategic choices under bounded rationality to maximize their respective interests.

Assumption 2: Decision-making Assumption for Pharmaceutical Companies. Pharmaceutical companies aim to maximize their total profits in each decision-making process. After considering compliance costs, illicit gains, the risk of penalties, and reputation damage, these companies will decide whether to adhere to health insurance regulations.

Assumption 3: Response Assumption for Third-Party Auditing Organizations. Third-Party Auditing Organizations base their actions on audit efficiency, potential rewards/subsidies, and the risk of fines. After evaluating their economic benefits and professional reputation, these agencies will decide whether to accept bribes from pharmaceutical companies.

Assumption 4: Behavioral Assumption for Medical Insurance Regulatory Agency. Medical Insurance Regulatory Agency adjust their regulatory strategies and costs based on the behaviors of pharmaceutical companies and Third-Party Auditing Organizations. Their goal is to maximize social welfare, ensure the safety of the health insurance fund, and promote compliant operations within the industry.

Assumption 5: Strategic Adjustment. During the game, each party adjusts its strategies based on the outcomes of the game and feedback received. For instance, if pharmaceutical companies frequently incur penalties for non-compliance, they may increase their compliance efforts and reduce violations. Similarly, if auditing agencies face severe penalties for misconduct, they may implement audit standards more rigorously.

## 4 Evolutionary model construction and analysis

### 4.1 Model construction

Set up the game model where pharmaceutical companies complying with health insurance regulations is denoted by x, non-compliance by 1-x, Third-Party Auditing Organizations accepting bribes by y, refusing bribes by 1-y, health insurance regulatory agencies enforcing strict regulation by z, and lenient regulation by 1-z, with the assumption that *x, y, z*∈(0, 1). Based on these assumptions and variable definitions, construct a mixed-strategy game payoff matrix involving pharmaceutical companies, third-party auditing organizations, and health insurance regulatory agencies as shown in Table 2.

**Table 2:** Mixed-strategy game payoff matrix for pharmaceutical companies, third-party auditing organizations, and health insurance regulatory agencies.

		**Third-party auditing organizations**	**Medical Insurance Regulatory Agency**	
			**Strict regulation (z)**	**Lenient regulation (1-z)**
Pharmaceutical Company	Compliance with Health Insurance Rules (x)	Accepts Bribes (y)	*W*_1_−*C*_2_+*R*_1_ *W*_3_−*E*_*t*_−*F*_*s*_−*E*_*s*_ −*C*_1_−*L*_2_+*N*_2_+*F*_*s*_	*W*_1_−*C*_2_+*R*_1_ *W*_3_−*E*_*t*_−*E*_*s*_ −*C*_1_−*L*_2_−*S*_*t*_
		Refuses Bribes (1-y)	*W*_1_−*C*_2_+*R*_1_ *W*_3_+*S*_*t*_−*E*_*t*_ −*C*_1_−*R*_1_−*S*_*t*_	*W*_1_−*C*_2_+*R*_1_ *W*_3_+*S*_*t*_−*E*_*t*_ −*R*_1_−*S*_*t*_
	Non-compliance with Health Insurance Rules (1-x)	Accepts Bribes (y)	*W*_1_−*C*_2_−*C*_3_−*F*_*h*_+*R*_2_ *W*_3_+*M*−*E*_*s*_−*F*_*s*_+*C*_3_ −*C*_1_−*L*_1_−*L*_2_+*N*_1_+*N*_2_+*F*_*h*_+*F*_*s*_	*W*_1_−*C*_2_−*C*_3_+*R*_2_ *W*_3_+*M*−*E*_*s*_+*C*_3_ −*C*_1_−*L*_1_−*L*_2_
		Refuses Bribes (1-y)	*W*_1_−*C*_2_−*C*_3_−*F*_*h*_ *W*_3_+*S*_*t*_−*E*_*t*_ −*C*_1_−*L*_1_+*N*_1_+*F*_*h*_	*W*_1_−*C*_2_−*C*_3_ *W*_3_+*S*_*t*_−*E*_*t*_ −*C*_1_−*L*_1_

### 4.2 Game model analysis

#### 4.2.1 The gains of pharmaceutical companies complying with the health care rules, not complying with the health care rules, and the average gains, respectively

$E_{X}={\left(W_{1}-C_{2}+R_{1}\right)}^{*}y^{*}z+{\left(W_{1}-C_{2}+R_{1}\right)}^{*}y^{*}\left(1-z\right)+{\left(W_{1}-C_{2}+R_{1}\right)}^{*}{\left(1-y\right)}^{*}z+{\left(W_{1}-C_{2}+R_{1}\right)}^{*}{\left(1-y\right)}^{*}\left(1-z\right)\;$

$E_{1-X}={\left(W_{1}-C_{2}-C_{3}-F_{h}+R_{2}\right)}^{*}y^{*}z+{\left(W_{1}-C_{2}-C_{3}+R_{2}\right)}^{*}y^{*}\left(1-z\right)+{\left(W_{1}-C_{2}-C_{3}-F_{h}\right)}^{*}{\left(1-y\right)}^{*}z+{\left(W_{1}-C_{2}-C_{3}\right)}^{*}{\left(1-y\right)}^{*}\left(1-z\right)\;$

$\bar{E}=xE_{x}+\left(1-x\right)E_{1-x}\;$

#### 4.2.2 The gains from accepting bribes, refusing bribes, and the average gain for third-party auditors are, respectively

$E_{y}={\left(W_{3}-E_{t}-F_{s}-E_{s}\right)}^{*}x^{*}z+{\left(W_{3}-E_{t}-E_{s}\right)}^{*}x^{*}\left(1-z\right)+{\left(1-x\right)}^{*}z^{*}\left(W_{3}+M-E_{s}-F_{s}+C_{3}\right)+{\left(W_{3}+M-E_{s}+C_{3}\right)}^{*}{\left(1-x\right)}^{*}\left(1-z\right)\;$

$E_{1-y}={\left(W_{3}+S_{t}-E_{t}\right)}^{*}x^{*}z+{\left(W_{3}+S_{t}-E_{t}\right)}^{*}x^{*}\left(1-z\right)+{\left(1-x\right)}^{*}z^{*}\left(W_{3}+S_{t}-E_{t}\right)+{\left(W_{3}+S_{t}-E_{t}\right)}^{*}{\left(1-x\right)}^{*}\left(1-z\right)\;$

$\bar{E}=yE_{y}+\left(1-y\right)E_{1-y}\;$

#### 4.2.3 The gains from strict and lax regulation by the health care regulator, as well as the average gains

$E_{z}={\left(-C_{1}-L_{2}+N_{2}+F_{s}\right)}^{*}x^{*}y+{\left(-C_{1}-L_{2}-S_{t}\right)}^{*}x^{*}\left(1-y\right)+{\left(-C_{1}-L_{1}-L_{2}+N1+N_{2}+F_{h}+F_{s}\right)}^{*}{\left(1-x\right)}^{*}y+{\left(-C_{1}-L_{1}+N_{1}+F_{h}\right)}^{*}{\left(1-x\right)}^{*}\left(1-y\right)\;$

$E_{z}={\left(-C_{1}-L_{2}+N_{2}+F_{s}\right)}^{*}x^{*}y+{\left(-C_{1}-L_{2}-S_{t}\right)}^{*}x^{*}\left(1-y\right)+{\left(-C_{1}-L_{1}-L_{2}+N1+N_{2}+F_{h}+F_{s}\right)}^{*}{\left(1-x\right)}^{*}y+{\left(-C_{1}-L_{1}+N_{1}+F_{h}\right)}^{*}{\left(1-x\right)}^{*}\left(1-y\right)\;$

$E_{1-z}=(-C_{1}-L_{2}-S_{t}{)}^{*}x^{*}y+(-R_{1}-S_{t}{)}^{*}x^{*}\left(1-y\right)+(-C_{1}-L_{1}-L_{2}{)}^{*}(1-x{)}^{*}y+(-C_{1}-L_{1}{)}^{*}(1-x{)}^{*}\left(1-\;y\right)$

$\bar{E}=zE_{z}+\left(1-z\right)E_{1-z}\;$

#### 4.2.4 The replicated dynamic equations for pharmaceutical companies, third-party auditors, and health insurance regulators

$F_{x}=-x^{*}{\left(x-1\right)}^{*}\left(C_{3}+R_{1}+F_{h}^{*}z-R_{2}^{*}y\right)\;$

$F_{y}=y^{*}{\left(y-1\right)}^{*}\left(E_{s}-C_{3}-E_{t}-M+S_{t}+C_{3}^{*}x+E_{t}^{*}x+F_{s}^{*}z+M^{*}x\right)\;$

$F_{z}=-z^{*}{\left(z-1\right)}^{*}\left(F_{h}+N_{1}-C_{1}^{*}x-F_{h}^{*}x+F_{s}^{*}y-N_{1}^{*}x+N_{2}^{*}y+C_{1}^{*}x^{*}y+S_{t}^{*}x^{*}y\right)\;$

### 4.3 Analysis of evolutionary system equilibrium

The strategic interactions among pharmaceutical companies, third-party auditing organizations, and health insurance regulatory agencies are continuously evolving. Therefore, by establishing a tripartite game model and deriving the replicator dynamic equations, the equilibrium points of this game can be calculated. The evolutionary process of the game involving the three parties is dynamic, with the probability of choosing any strategy being time-dependent. According to the stability principles of differential equations, when all dynamic equations reach zero, it signifies that the entire dynamic system will tend toward stability. Thus, by constructing the replicator dynamic equations for the tripartite game model and setting *F*_*x*_ = 0,*F*_*y*_ = 0,*F*_*z*_ = 0the evolutionary equilibrium points of the game can be determined. Specifically, let:

$F_{x}=-x^{*}{\left(x-1\right)}^{*}\left(C_{3}+R_{1}+F_{h}^{*}z-R_{2}^{*}y\right)=0\;$

$F_{y}=y^{*}{\left(y-1\right)}^{*}\left(E_{s}-C_{3}-E_{t}-M+S_{t}+C_{3}^{*}x+E_{t}^{*}x+F_{s}^{*}z+M^{*}x\right)=\;0\;$

$F_{z}=-z^{*}{\left(z-1\right)}^{*}\left(F_{h}+N_{1}-C_{1}^{*}x-F_{h}^{*}x+F_{s}^{*}y-N_{1}^{*}x+N_{2}^{*}y+C_{1}^{*}x^{*}y+S_{t}^{*}x^{*}y\right)=\;0\;$

Based on Selten's research findings in non-cooperative game theory, under conditions of asymmetric information, evolutionarily stable strategies are pure strategies. Therefore, it is necessary only to discuss the asymptotic stability of the eight local equilibrium points *E*_1_(0, 0, 0), *E*_2_(1, 0, 0), *E*_3_(0, 1, 0), *E*_4_(0, 0, 1), *E*_5_(1, 1, 0), *E*_6_(1, 1, 0), *E*_7_(0, 1, 1), *E*_8_(1, 1, 1) that satisfy *F*_*x*_ = 0, *F*_*y*_ = 0, *F*_*z*_ = 0.Using the replicator dynamics equations of the three parties, the Jacobian matrix of the evolutionary game system can be obtained, which facilitates the analysis of the stability of the equilibrium points:

$J=\left[\begin{array}{ccc} \frac{\partial F_{x}}{\partial x} & \frac{\partial F_{x}}{\partial y} & \frac{\partial F_{x}}{\partial z}\\ \frac{\partial F_{y}}{\partial x} & \frac{\partial F_{y}}{\partial y} & \frac{\partial F_{y}}{\partial z}\\ \frac{\partial F_{z}}{\partial x} & \frac{\partial F_{z}}{\partial y} & \frac{\partial F_{z}}{\partial z} \end{array}\right]=\left[\begin{array}{ccc} J_{11} & J_{12} & J_{13}\\ J_{21} & J_{22} & J_{23}\\ J_{31} & J_{32} & J_{33} \end{array}\right]\;$

$J_{11}=-x^{*}\left(C_{3}+\;R_{1}+\;F_{h}^{*}z-R_{2}^{*}y\right)\;-\;{\left(x\;-\;1\right)}^{*}\left(C_{3}+\;R_{1}+\;F_{h}^{*}z-R_{2}^{*}y\right)\;$

$J_{12}=R_{2}^{*}x^{*}\left(x-\;1\right)\;$

$J_{13}=\;-F_{h}^{*}x^{*}\left(x\;-1\right)\;$

$J_{21}=y^{*}{\left(y\;-\;1\right)}^{*}\left(C_{3}\;+\;E_{t}\;+\;M\right)\;$

$J_{22}=y^{*}\left(E_{s}\;-\;C_{3}\;-\;E_{t}\;-\;M\;+\;S_{t}\;+\;C_{3}^{*}x\;+\;E_{t}^{*}x\;+\;F_{s}^{*}z\;+\;M^{*}x\right)\;+\;{\left(y\;-\;1\right)}^{*}\left(E_{s}-\;C_{3}\;-\;E_{t}\;-\;M\;+\;S_{t}+C_{3}^{*}x\;+\;E_{t}^{*}x\;+\;F_{s}^{*}z\;+\;M^{*}x\right)\;$

$J_{23}=\;F_{s}^{*}y^{*}\left(y-1\right)\;$

$J_{31}=z^{*}{\left(z-1\right)}^{*}\left(C_{1}+F_{h}+N_{1}-C1^{*}y-S_{t}^{*}y\right)$

$J_{32}=-z^{*}{\left(z-1\right)}^{*}\left(F_{s}+N_{2}+C_{1}^{*}x+S_{t}^{*}x\right)\;$

$J_{33}=-{\left(z-1\right)}^{*}\left(F_{h}+N_{1}-C_{1}^{*}x-F_{h}^{*}x+F_{s}^{*}y-N_{1}^{*}x+N_{2}^{*}y+C_{1}^{*}x^{*}y+S_{t}^{*}x^{*}y\right)-z^{*}\left(F_{h}+N_{1}-C_{1}^{*}x-F_{h}^{*}x+\;F_{s}^{*}y-N_{1}^{*}x+N_{2}^{*}y+C_{1}^{*}x^{*}y+S_{t}^{*}x^{*}y\right)$

Using the Lyapunov method, it is known that the stability of a differential system can be determined by the signs of the eigenvalues of the equilibrium points. An equilibrium point is considered an evolutionarily stable strategy (asymptotically stable) if all its eigenvalues (roots) are negative. The eight pure strategy points are each substituted into the Jacobian matrix in turn, and the eigenvalues of the equilibrium points are calculated as shown in Table 3.

**Table 3:** Equilibrium points and their eigenvalues.

**Equilibrium point**	**Eigenvalues of the Jacobian matrix**			**Stability conclusion**
	**λ**_**1**_	**λ**_**2**_	**λ**_**3**_	
*E*_1_(0, 0, 0)	*C*_3_−*R*_1_	*C*_3_−*E*_*s*_+*E*_*t*_+*M*−*S*_*t*_	*F*_*h*_+*N*_1_	Unstable
*E*_2_(1, 0, 0)	−*C*_3_−*R*_1_	−*E*_*s*_−*S*_*t*_	−*C*_1_	ESS (Evolutionarily Stable Strategy)
*E*_3_(0, 1, 0)	*C*_3_+*R*_1_−*R*_2_	*E*_*s*_−*C*_3_−*E*_*t*_−*M*+*S*_*t*_	*F*_*h*_+*F*_*s*_+*N*_1_+*N*_2_	Unstable
*E*_4_(0, 0, 1)	*C*_3_+*F*_*h*_+*R*_1_	*C*_3_−*E*_*s*_+*E*_*t*_−*F*_*s*_+*M*−*S*_*t*_	− *F*_*h*_−*N*_1_	Unstable
*E*_5_(1, 1, 0)	_*R*_2_−*R*_1_−*C*3_	*E*_*s*_+*S*_*t*_	*F*_*s*_+*N*_2_+*S*_*t*_	Unstable
*E*_6_(1, 0, 1)	−_*C*_3_−*F*_*h*_−*R*1_	− *E*_*s*_−*F*_*s*_−*S*_*t*_	*C*_1_	Unstable
*E*_7_(0, 1, 1)	*C*_3_+*F*_*h*_+*R*_1_−*R*_2_	*E*_*s*_−*C*_3_−*E*_*t*_+*F*_*s*_−*M*+*S*_*t*_	−*F*_*h*_−*F*_*s*_−*N*_1_−*N*_2_	Unstable
*E*_8_(1, 1, 1)	_*R*_2_−*F*_*h*_−*R*_1_−*C*3_	*E*_*s*_+*F*_*s*_+*S*_*t*_	− *F*_*s*_−*N*_2_−*S*_*t*_	Unstable

## 5 Numerical simulation analysis

### 5.1 Evolutionarily stable strategy

In the tripartite game of China's pharmaceutical industry, the equilibrium point *E*_2_(1, 0, 0), represents a scenario where pharmaceutical companies comply with health insurance regulations, third-party auditing organizations reject bribes, and health insurance regulatory agencies adopt a lenient supervision approach. The formation of this optimal strategy is based on a comprehensive consideration of multiple factors.

Firstly, the rationale for pharmaceutical companies to comply with health insurance rules lies in the comparison between normal income under compliance (*W*_1_) and the compensations received from health insurance (*R*_1_), which outweigh the high risks and potential losses (*C*_3_,*F*_*h*_) associated with non-compliant activities. Although higher income (*W*_2_) and illegal health insurance benefits (*R*_2_) might be obtained through non-compliance, the uncertainty and high risks associated with these gains make compliance a more stable and reliable operation strategy. Secondly, the strategy of third-party auditing organizations to refuse bribes is based on a comparison between their regular earnings (*W*_3_) and the costs of audits (*E*_*t*_). While accepting bribes could bring additional income (M), it also comes with substantial penalties for violations (*F*_*s*_) and potential losses/risk (*E*_*s*_). Thus, refusing bribes ensures long-term stable income and maintains the reputation of third-party auditing organizations. Lastly, the reason for Medical Insurance Regulatory Agency to opt for lenient supervision is that it can reduce the costs of oversight (*C*_1_) and avoid excessive market intervention. Moreover, lenient supervision can encourage compliant behavior among third-party auditors and pharmaceutical companies through incentives such as financial subsidies/awards (*S*_*t*_), rather than solely relying on punitive measures.

Therefore, the formation of equilibrium point *E*_2_(1, 0, 0) is based on the combined influences of pharmaceutical companies seeking stable revenues, third-party auditors aiming for long-term robust operations, and health insurance regulators pursuing efficient oversight and market incentives. This strategic choice helps to reduce corruption in the pharmaceutical industry, balance the interests of all parties, and promote the industry's healthy development. However, real-world conditions may be influenced by various factors, thus further research and empirical analysis are required to validate the effectiveness and applicability of this equilibrium point.

### 5.2 Parameter sensitivity analysis

To validate the effectiveness of the evolutionary stability analysis, numerical values were assigned to the model, and numerical simulations were conducted using MATLAB [Version 9.9.0 (R2020b), The MathWorks Inc., Natick, MA, USA].

#### 5.2.1 Impact of regulatory costs (***C***_**1**_) on the evolution of the tripartite game

**Figure 2:**
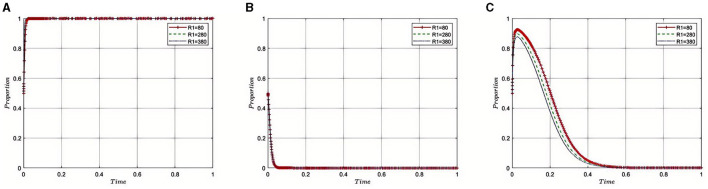
Sensitivity analysis of regulatory costs on evolutionary strategies. **(A–C)** respectively represent pharmaceutical companies, Third-Party Auditing Organizations, and Medical Insurance Regulatory Agency. The same applies below.

Under the equilibrium conditions of point *E*_2_(1, 0, 0), assume that *C*_1_ = 15, 20, 30, as illustrated in Figure 2. The simulation results indicate that as regulatory costs increase, the Medical Insurance Regulatory Agency may face resource and fiscal pressures, leading to a propensity toward more lenient regulatory strategies. This relaxation in supervision is partly due to the high costs making enforcement challenging, as the department might not be able to sustain the resources needed for strict regulation continuously. To maintain effective oversight with limited resources, the Medical Insurance Regulatory Agency might choose to collaborate with third-party auditing organizations, leveraging their expertise and capabilities to share the responsibilities of regulation. This strategy can alleviate some of the pressures of regulatory costs while maintaining the effectiveness of supervision. However, it also presents challenges in ensuring the independence of third-party auditors and avoiding conflicts of interest. Therefore, the Medical Insurance Regulatory Agency needs to find a balance between lenient regulation and collaboration with third parties, ensuring the quality and fairness of supervision while effectively managing regulatory costs.

#### 5.2.2 Impact of the medical insurance regulatory agency's recovery of fines(***N***_**1**_) from pharmaceutical companies on the evolution of the tripartite game

**Figure 3:**
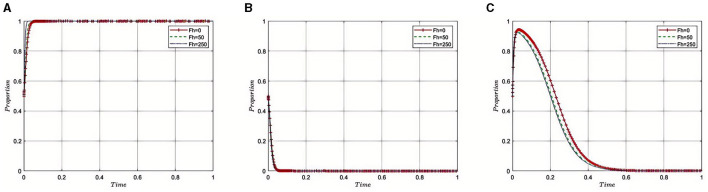
**(A–C)** Influence of fines recovered from pharmaceutical companies on compliance behavior.

Under the equilibrium conditions of point *E*_2_(1, 0, 0), assume that *N*_1_= 180, 250, 450, as depicted in Figure 3. The simulation results show that the magnitude of *N*_1_ directly determines the behavioral strategy of pharmaceutical companies. With a lower N1, where the amount recovered by the Medical Insurance Regulatory Agency from violations is minimal, the penalties imposed on pharmaceutical companies appear more lenient. This scenario may lead pharmaceutical companies to adopt high-risk strategies, such as violating health insurance rules to increase profits, thereby exacerbating non-compliant behavior within the industry. However, as N1 increases, indicating that a larger amount of illicit gains is being recovered, it reflects that the Medical Insurance Regulatory Agency is intensifying penalties for violations. This stricter regulatory approach compels pharmaceutical companies to comply with the rules, reducing non-compliant activities and contributing to enhanced overall governance of the industry and public health safety.

#### 5.2.3 Impact of the medical insurance regulatory agency's recovery of fines(***N***_**2**_) from third-party auditing organizations on the evolution of the tripartite game

**Figure 4:**
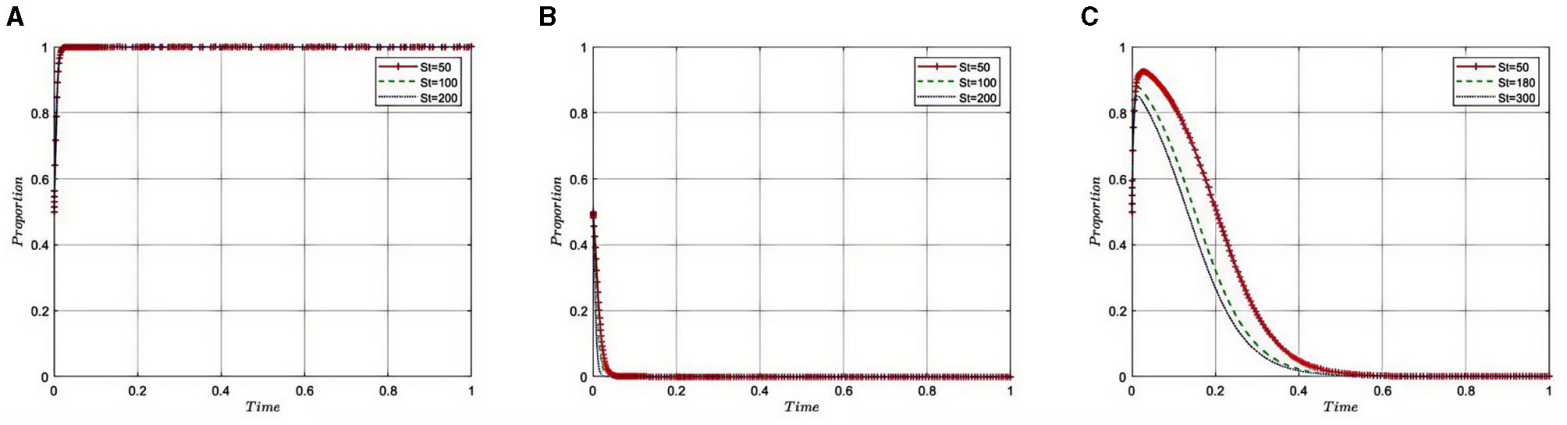
**(A–C)** Effect of fines recovered from third-party auditing organizations on auditing integrity.

Under the equilibrium conditions at point *E*_2_(1, 0, 0), assume that *N*_2_= 150, 200, 300, as illustrated in Figure 4. The simulation results demonstrate that the magnitude of *N*_2_ directly affects the behavioral choices of Third-Party Auditing Organizations. When *N*_2_ is low, indicating a smaller amount recovered for violations, it suggests that the health insurance regulatory oversight and penalties on auditing agencies are lenient. In such cases, auditing agencies might be inclined to take risks, such as accepting bribes from pharmaceutical companies, thereby increasing non-compliant behavior within the pharmaceutical industry. Conversely, higher values of *N*_2_ signify stricter supervision and more severe penalties, leading auditing agencies to favor compliant behaviors and reduce instances of accepting bribes. This contributes to enhancing the transparency and reputation of the entire pharmaceutical industry, promoting its healthy development. Therefore, by adjusting the amount of fines recovered, the Medical Insurance Regulatory Agency can effectively control compliance within the pharmaceutical industry, influencing internal strategic choices and behavioral patterns, and thus fostering the industry's health and sustainable development.

#### 5.2.4 Impact of fines imposed by medical insurance regulatory agency's on pharmaceutical companies (***F***_***h***_) on the evolution of the tripartite game

**Figure 5:**
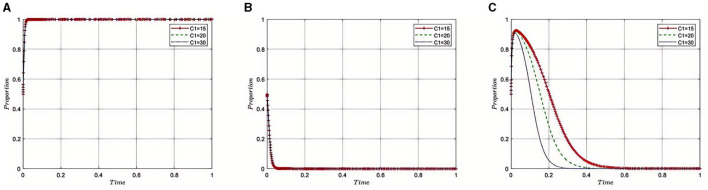
**(A–C)** Impact of penalties on pharmaceutical companies' regulatory compliance.

Under the equilibrium conditions at point *E*_2_(1, 0, 0), assume that *F*_*h*_= 0, 150, 250, the simulation evolution is depicted in Figure 5. The simulation results indicate that as the fines imposed by the Medical Insurance Regulatory Agencys on pharmaceutical companies increase, indicating stricter penalties for regulatory violations, there is a corresponding motivation for pharmaceutical companies to adhere to industry regulations and reduce non-compliant activities. Initially, as fines are increased, the regulatory department may observe a significant enhancement in industry compliance, potentially leading to an intensification of regulatory efforts to ensure effective implementation of the new fining system. However, once fines reach a certain level, diminishing returns may occur, where further increases in fines no longer significantly enhance compliance. In such cases, the regulatory department may choose to maintain fines at this level to continue effective oversight while avoiding negative impacts on industry development. This strategy aims to find a reasonable balance between the level of fines and regulatory intensity, ensuring the health and sustainable development of the industry, while safeguarding market stability and public interests.

#### 5.2.5 Impact of health insurance compensation (***R***_**1**_) on pharmaceutical companies' compliance on the evolution of the tripartite game

**Figure 6:**
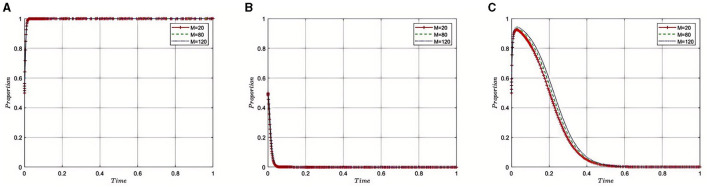
**(A–C)** Effect of health insurance compensation on pharmaceutical companies' compliance.

Under the equilibrium conditions at point *E*_2_(1, 0, 0), assume that *R*_1_= 80, 280, 380, the simulation evolution is shown in Figure 6. The simulation results indicate that increased health insurance compensation for compliance encourages pharmaceutical companies to adhere more strictly to regulatory standards. At lower compensation levels (*R*_1_ = 80), the incentive might not be sufficient to deter companies from non-compliance if the penalties for non-compliance are relatively low. However, as compensation increases (*R*_1_ = 280 and 380), it becomes economically advantageous for companies to comply with health insurance regulations, leading to a substantial increase in compliance rates.

This rise in compensation makes it financially beneficial for companies to invest in the necessary adjustments to align with regulations, outweighing the potential short-term gains from non-compliance. The strategic interactions in the tripartite game suggest that higher compliance driven by better compensation could reduce the need for harsh penalties and intense monitoring by the Medical Insurance Regulatory Agency, promoting a more cooperative and less adversarial relationship within the industry. However, the model also indicates a threshold beyond which increases in compensation do not yield proportional increases in compliance. This plateau effect suggests that beyond a certain point, additional compensation may not be cost-effective for the health insurance system.

#### 5.2.6 Impact of illicit health insurance benefits (***R***_**2**_) on pharmaceutical companies on the evolution of the tripartite game

**Figure 7:**
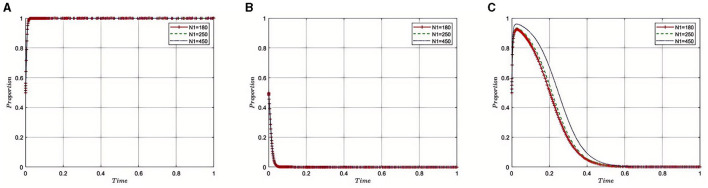
**(A–C)** Impact of illicit benefits on non-compliance by pharmaceutical companies.

Under the equilibrium conditions at point *E*_2_(1, 0, 0), assume that *R*_2_ = 100, 200, 300, as depicted in Figure 7. The simulation results indicate that as *R*_2_ increases, reflecting greater benefits obtained by pharmaceutical companies through non-compliance, the Medical Insurance Regulatory Agency may face a need to intensify its supervisory efforts to address the growing prevalence of non-compliance and fraudulent practices within the industry. In this context, the regulatory department might adopt stricter monitoring and punitive measures to ensure that pharmaceutical companies adhere to industry regulations and reduce non-compliant activities. Concurrently, the role of third-party auditing firms becomes increasingly critical. As the illicit gains of pharmaceutical companies rise, the Medical Insurance Regulatory Agency may rely more heavily on these agencies for thorough and professional auditing to ensure the transparency and compliance of financial and operational practices of pharmaceutical companies. Therefore, in the context of increased non-compliant gains by pharmaceutical companies, both the Medical Insurance Regulatory Agency and Third-Party Auditing Organizations need to strengthen their collaboration and adopt stricter measures to maintain the integrity of the pharmaceutical industry and public interests, effectively reducing non-compliant activities and ensuring the safety of health insurance funds.

#### 5.2.7 Impact of financial subsidies/awards (***S***_***t***_) provided by the medical insurance regulatory agency to third-party auditing organizations on the evolution of the tripartite game

**Figure 8:**
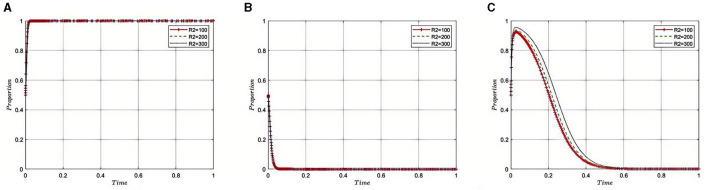
**(A–C)** Influence of financial incentives for auditing organizations on compliance.

Under the equilibrium conditions at point *E*_2_(1, 0, 0), assume that *S*_*t*_ = 50, 100, 200, as illustrated in Figure 8. The simulation results indicate that as these subsidies or awards increase, Third-Party Auditing Organizations receive greater incentives to conduct more stringent and detailed audits, which enhances their work quality and efficiency. Such financial incentives are crucial for improving the transparency and compliance of pharmaceutical companies' operations. However, once these subsidies or awards reach a certain level, diminishing returns may occur, suggesting that further increases in subsidies or awards do not significantly enhance auditing outcomes. Therefore, the Medical Insurance Regulatory Agency might choose to stabilize subsidies or awards at a certain level to maintain reasonable incentives for auditing agencies while avoiding unnecessary financial burdens. In this way, the Medical Insurance Regulatory Agency can effectively motivate Third-Party Auditing Organizations to participate, enhance the regulatory quality of the pharmaceutical industry, and ensure the efficiency and rationality of fiscal expenditures.

#### 5.2.8 Impact of additional earnings (*M*) obtained by third-party auditing organizations from pharmaceutical companies' bribes on the evolution of the tripartite game

**Figure 9:**
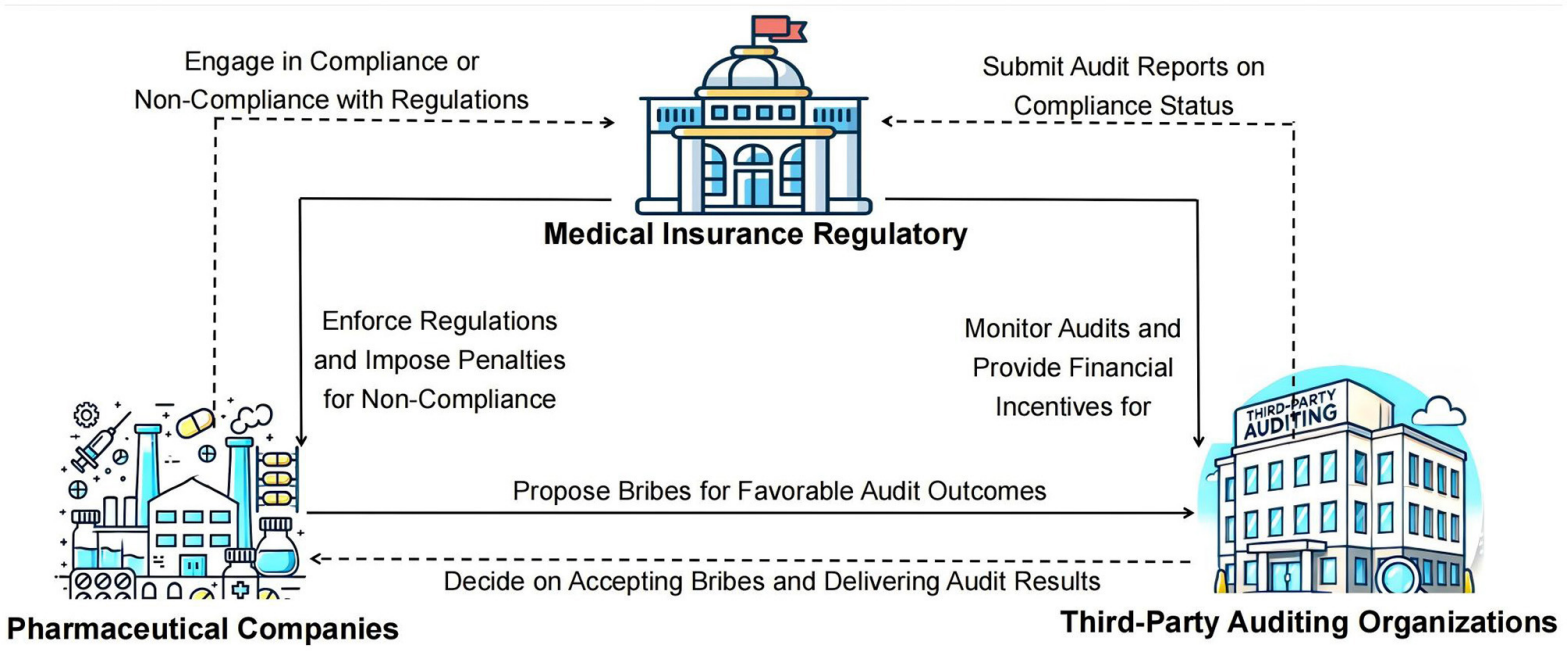
Effect of bribes on auditing organizations' strategic choices.

Under the equilibrium conditions at point *E*_2_(1, 0, 0), assume that *M* = 20, 80, 120, as depicted in Figure 9. The simulation results show that while accepting bribes and gaining additional income may provide short-term economic benefits for auditing agencies, such practices increase the opacity and non-compliance within the industry, thereby damaging the independence and credibility of the auditing agencies. This leads to the Medical Insurance Regulatory Agency implementing stronger regulatory measures and imposing severe penalties on the involved auditing agencies and pharmaceutical companies. Auditing agencies must weigh the economic benefits against the potential regulatory risks when pursuing additional income. If bribery is detected, it not only severely damages the reputation of the auditing agencies but also may trigger forceful interventions by the Medical Insurance Regulatory Agency, affecting the overall health and stability of the pharmaceutical industry. Therefore, to ensure the long-term stability and compliance of the pharmaceutical industry, auditing agencies need to consider the long-term impact on the industry and maintain their professional ethical standards while contemplating short-term benefits.

### 5.3 Summary

This study conducts an in-depth analysis of the parameter sensitivity in the tripartite game within the pharmaceutical industry, highlighting the specific impacts of various regulatory factors on the strategic choices of pharmaceutical companies, Third-Party Auditing Organizations, and Medical Insurance Regulatory Agency (Table 4). Through numerical simulation analysis, the study reveals how factors such as regulatory costs, recovery of illicit amounts, penalty severity, health insurance compensation, and fiscal incentives influence the behavioral patterns of the parties involved. For instance, low regulatory costs may encourage pharmaceutical companies to adopt riskier strategies, while high recovery of illicit amounts significantly strengthens the incentives for rule compliance. Additionally, the analysis examines how fiscal incentives can promote more rigorous and thorough auditing by auditing agencies, and how high penalties can enhance compliance among all parties. These findings provide important bases for regulatory strategies in the pharmaceutical industry, emphasizing the crucial role of effective regulatory policies in balancing the interests within the industry.

**Table 4:** Summary of parameter sensitivity in the tripartite game within the pharmaceutical industry.

**Factor**	**Impact on pharmaceutical companies**	**Impact on third-party auditing organizations**	**Impact on medical insurance regulatory agency**
Regulatory costs (*C*_1_)	Low regulatory costs may prompt pharmaceutical companies to adopt high-risk strategies.	Reduced regulatory costs could ease the auditing burden.	Increased regulatory costs may lead to a shift toward more flexible and efficient supervision strategies.
Recovery of violation amounts (*N*_1_, *N*_2_)	High recovery amounts enhance compliance.	When violation amounts are high, reduce bribery and promote compliance.	High recovery amounts indicate strong punitive measures by regulatory bodies, promoting industry compliance.
Penalties (*F*_*h*_)	Increased penalties encourage compliance with industry regulations.	The risk of accepting bribes is related to penalties; high penalties promote compliance.	Strengthening penalties enhances regulatory effectiveness and ensures enforcement of regulations.
Health insurance compensation (*R*_1_, *R*_2_)	High compensation under compliance encourages compliance; high illicit gains increase the risk of violations.	Higher compensation levels for pharmaceutical companies can reduce the auditing workload by promoting rule compliance.	Higher compensation levels may require stronger regulatory measures.
Financial subsidies/rewards (*S*_*t*_)	Financial incentives can encourage companies to adopt more compliant business strategies.	Financial incentives promote more rigorous and detailed auditing.	Appropriate financial incentives can improve auditing quality and efficiency, while controlling costs.
Bribery benefits (*M*)	Accepting bribes may increase short-term profits but raises long-term legal and reputational risks, affecting sustainability.	Balancing the short-term benefits and long-term risks of accepting bribes.	Strengthening oversight of auditing organizations to ensure independence and credibility.

## 6 Research findings and discussion

### 6.1 Research findings

This study, grounded in the theoretical framework of game theory, delves into the complex interactions among pharmaceutical companies, third-party auditing organizations, and Medical Insurance Regulatory Agencys within the Chinese pharmaceutical industry. By constructing an evolutionary game model, the study reveals the dynamic evolution of strategy choices under economic incentives and penalty mechanisms, as well as the impact of these choices on the security of health insurance funds and industry governance. The model analysis indicates that the optimal strategy equilibrium is achieved when pharmaceutical companies comply with health insurance regulations, third-party auditors reject bribes, and Medical Insurance Regulatory Agency implement lenient supervision. This strategic equilibrium reduces corruption in the pharmaceutical industry, balances the interests of all parties, and promotes the healthy development of the sector. Additionally, numerical simulation analysis of key parameters in the model shows that factors such as regulatory costs, recovery of improper charges, and fines imposed on pharmaceutical companies significantly influence the equilibrium state. Particularly, the interplay of factors like the compliance costs vs. the risk of non-compliance benefits for pharmaceutical companies, the professional reputation vs. economic benefits for auditing organizations, and the goal of maximizing social welfare for regulatory bodies, determine the ultimate equilibrium strategy. Therefore, pharmaceutical companies, in their pursuit of maximizing benefits, need to weigh the risks of compliance costs against non-compliance gains; third-party auditors make choices between fulfilling their duties and accepting bribes, considering their economic benefits and professional reputation; and Medical Insurance Regulatory Agencys adjust their strategies between strict and lenient supervision, aiming to maximize social welfare.

### 6.2 Discussion and recommendations

In this study, we examined the corruption issues in the Chinese pharmaceutical industry through an evolutionary game model, analyzing the interactions among pharmaceutical companies, third-party auditing organizations, and Medical Insurance Regulatory Agencys. In light of China's policy environment, national conditions, and the findings of this analysis, we offer the following discussions and recommendations:

#### 6.2.1 Enhancing policy support and legal framework construction

First and foremost, it is essential to strengthen policy support and construct a robust legal framework. The government should enhance its support by developing and implementing specialized policies targeted at unique issues within the pharmaceutical industry, with a focus on drug quality control, market price regulation, and the standardization of pharmaceutical advertising. Moreover, updating and improving the current legal framework to accommodate industry growth and emerging challenges, such as online drug sales and international pharmaceutical collaborations, is crucial for maintaining industry order. Concurrently, the government must intensify law enforcement efforts to ensure effective implementation of laws and policies, impose stringent legal sanctions on violations, and thereby increase the cost of non-compliance. Establishing a diverse regulatory system that includes government oversight, industry self-regulation, and public participation will enhance regulatory efficiency and transparency, thereby fostering the healthy development of the industry and safeguarding public health and safety.

#### 6.2.2 Enhancing compliance and self-regulation in pharmaceutical companies

One of the main challenges facing the Chinese pharmaceutical industry is ensuring that pharmaceutical companies comply with regulations and industry standards in a rapidly developing market. The government should increase regulatory efforts and establish clear legal frameworks and guidelines to prevent and punish improper market conduct. Simultaneously, pharmaceutical companies are encouraged to establish comprehensive internal compliance systems and strengthen their sense of corporate social responsibility. By enhancing transparency and ethical standards, companies can protect consumer interests and safeguard their own long-term development interests. Additionally, encouraging pharmaceutical companies to conduct self-monitoring, regularly publish compliance reports, and undergo public and regulatory scrutiny will further strengthen the industry's self-regulatory mechanisms.

#### 6.2.3 Enhancing the independence and professional capabilities of third-party auditing organizations

Third-party auditing organizations play a critical role in the regulatory system of the pharmaceutical industry, and their independence and professional capabilities are directly related to the effectiveness of regulation. Therefore, it is crucial to strengthen the industry entry and oversight management of these organizations. It is recommended to establish stricter auditing organization accreditation standards and regulatory systems to ensure their independence and objectivity during the audit process, avoiding conflicts of interest. Additionally, enhancing the professional skills and ethical standards of auditors through regular training and assessments is essential to ensure they can adapt to the rapid changes and complexities of the pharmaceutical industry. Strengthening the supervision of audit quality is also vital to ensure the accuracy and reliability of audit results.

#### 6.2.4 Optimizing regulatory strategies and technological applications of health insurance regulatory agencies

Health insurance regulatory agencies should consider the industry's specificities and the market's dynamic changes when developing and implementing regulatory strategies. It is recommended to adopt more flexible and efficient regulatory methods, such as using big data and artificial intelligence technologies for risk assessment and predictive analysis, to achieve precise regulation. Additionally, the government should strengthen the supervision of health insurance funds to prevent misuse and fraudulent activities, ensuring the rational allocation and utilization of resources. Meanwhile, appropriately relaxed regulatory strategies can reduce industry burdens, encourage enterprises to voluntarily comply with industry norms, and foster a positive industry atmosphere.

#### 6.2.5 Promoting multi-party cooperation and information sharing mechanisms

It is advisable to foster cooperation among governments, enterprises, and third-party institutions and establish effective information sharing mechanisms. This not only helps enhance regulatory efficiency but also strengthens the industry's self-regulatory capabilities. The government can facilitate this by creating an industry database to collect and analyze operational data from the pharmaceutical sector, identify potential issues promptly, and take appropriate actions. Encouraging communication and collaboration among pharmaceutical companies, auditing organizations, and related institutions is also essential to maintain industry order. Through the joint efforts of multiple parties, it is possible to combat corruption more effectively and promote the healthy development of the pharmaceutical industry.

## 7 Conclusion

This paper has conducted a comprehensive analysis of policy, oversight, and corruption issues in China's pharmaceutical industry through the development of an evolutionary game model. It particularly focused on the strategic interactions among pharmaceutical companies, third-party auditing organizations, and Medical Insurance Regulatory Agency, and their impact on industry corruption. The findings indicate that under reasonable economic incentives and regulatory constraints, these key stakeholders can form effective anti-corruption mechanisms to jointly promote the health and sustainable development of the pharmaceutical industry. The analysis also highlighted the importance of cooperative mechanisms and information sharing in improving pharmaceutical industry governance, emphasizing that policymakers need to consider market behaviors and economic factors when adjusting regulatory strategies. However, the limitations of this study include the model's simplifications, which may not fully capture the actual complex dynamics within the industry, such as rapid changes in policies and market environments; additionally, data acquisition and processing pose challenges that may limit the universality and precision of the model's conclusions. Future research directions will focus on further refining and deepening the model and theory, including considering more practical factors such as policy changes, technological innovations, and market competition, to enhance the model's applicability and predictive power. Moreover, broader use of big data and artificial intelligence technologies will be employed for in-depth analysis, improving the precision and efficiency of data processing to provide a more comprehensive and profound perspective for the healthy development of the pharmaceutical industry.

## Data availability statement

The raw data supporting the conclusions of this article will be made available by the authors, without undue reservation.

## Author contributions

XW: Conceptualization, Data curation, Funding acquisition, Methodology, Project administration, Supervision, Visualization, Writing – original draft, Writing – review & editing. TZ: Conceptualization, Project administration, Supervision, Writing – original draft, Writing – review & editing. HG: Conceptualization, Funding acquisition, Investigation, Project administration, Resources, Visualization, Writing – original draft, Writing – review & editing. JL: Conceptualization, Data curation, Formal analysis, Methodology, Software, Visualization, Writing – original draft, Writing – review & editing. BW: Formal analysis, Methodology, Project administration, Validation, Writing – review & editing, Writing – original draft. BC: Formal analysis, Project administration, Supervision, Validation, Writing – original draft, Writing – review & editing. SZ: Project administration, Validation, Writing – original draft, Writing – review & editing.

## Footnotes

^1^ https://www.samr.gov.cn/zw/zfxxgk/fdzdgknr/fgs/art/2023/art_3737890d856a4e44a8ea07c50c90c116.html

^2^ https://www.samr.gov.cn/zw/zfxxgk/fdzdgknr/fgs/art/2023/art_f0fae9eb3a684fc39e84d89eabfc2caa.html

## References

[1] Thorley M Fulda A. The importance of leverage in glaxosmithkline's china engagement: a revelatory case study. J Curr Chin Affairs. (2020) 49:233–54. 10.1177/1868102620931862

[2] Fu H Lai Y Li Y Zhu Y Yip W. Understanding medical corruption in China: a mixed-methods study. Health Policy Plan. (2023) 38:496–508. 10.1093/heapol/czad015 36798965

[3] Hsiao A Vogt V Quentin W. Effect of corruption on perceived difficulties in healthcare access in sub-Saharan Africa. PLoS ONE. (2019) 14:e0220583. 10.1371/journal.pone.0220583 31433821 PMC6703670

[4] Mackey TK Cuomo RE. An interdisciplinary review of digital technologies to facilitate anti-corruption, transparency and accountability in medicines procurement. Glob Health Action. (2020) 13:1695241. 10.1080/16549716.2019.1695241 32194014 PMC7170358

[5] Gotzsche P. (2019). Deadly Medicines and Organised Crime: How Big Pharma has Corrupted Healthcare. Boca Raton, FL: CRC Press.

[6] Onwujekwe O Orjiakor CT Hutchinson E Mckee M Agwu P Mbachu C. Where do we start? Building consensus on drivers of health sector corruption in Nigeria and ways to address it. Int J Health Policy Manage. (2020) 9:286. 10.15171/ijhpm.2019.128 32613800 PMC7444438

[7] Cosgrove L Mintzes B Bursztajn HJD Ambrozio G Shaughnessy AF. Industry effects on evidence: a case study of long-acting injectable antipsychotics. Accountab Res. (2022) 1–12. 10.1080/08989621.2022.2082289 35634753

[8] Arnold DG Stewart OJ Beck T. Financial penalties imposed on large pharmaceutical firms for illegal activities. JAMA. (2020) 324:1995–7. 10.1001/jama.2020.18740 33201196 PMC7672515

[9] Feldman Y Gauthier R Schuler T. Curbing misconduct in the pharmaceutical industry: Insights from behavioral ethics and the behavioral approach to law. J Law, Med Ethics. (2013) 41:620–8. 10.1111/jlme.12071 24088152

[10] Gagnon M. Corruption of pharmaceutical markets: addressing the misalignment of financial incentives and public health. J Law, Med Ethics. (2013) 41:571–80. 10.1111/jlme.12066 24088147

[11] Spinney L. Drugs, money and misleading evidence. Nature. (2020) 583:26–28. 10.1038/d41586-020-01911-7

[12] Kohler JC Dimancesco D. The risk of corruption in public pharmaceutical procurement: how anti-corruption, transparency and accountability measures may reduce this risk. Glob Health Action. (2020) 13:1694745. 10.1080/16549716.2019.1694745 32194011 PMC7170361

[13] Ariyanto D Jhuniantara I. Fraudulent financial statements in pharmaceutical companies: fraud pentagon theory perspective. J Legal Ethical & Regul. (2021) 4:6. 10.5267/j.ac.2021.5.009

[14] Zhou M Qu S Zhao L Kong N Campy KS Wang S. Trust collapse caused by the Changsheng vaccine crisis in China. Vaccine. (2019) 37:3419–25. 10.1016/j.vaccine.2019.05.020 31097351

[15] Huang C. China's practical wisdom: Assumption of liability for endangering public health in bankruptcy proceedings—a case study of the Changchun Changsheng Biotechnology vaccine incident and the Johnson & Johnson baby powder incident. Front Public Health. (2022) 10:1003330. 10.3389/fpubh.2022.1003330 36438243 PMC9682495

[16] Central Commission for Discipline Inspection and National Supervisory Commission. Guangdong Provincial Health Commission's Former Party Member and Deputy Director, and Former Director of the Provincial Administration of Traditional Chinese Medicine Xu Qingfeng Expelled from Party and Public Office. In: Central Commission for Discipline Inspection and National Supervisory Commission. (2024). Available at: https://www.ccdi.gov.cn/yaowenn/202404/t20240422_343089_m.html (accessed July 20, 2024).

[17] Hua X. Competitiveness of the Chinese Pharmaceutical Industry: Environment, Drivers and Strategies. Manchester: The University of Manchester (United Kingdom). (2019).

[18] Pasculli L Ryder N. Corruption in the Global Era: Causes, Sources and Forms of Manifestation. London: Routledge. (2019).

[19] Hua Y Lu J Bai B Zhao H. Can the profitability of medical enterprises Be improved after joining China's centralized drug procurement? A difference-in-difference design. Front Public Health. (2022) 9:809453. 10.3389/fpubh.2021.809453 35178375 PMC8843945

[20] Yip W Fu H Chen AT Zhai T Jian W Xu R. 10 years of health-care reform in China: progress and gaps in Universal Health Coverage. Lancet. (2019) 394:1192–204. 10.1016/S0140-6736(19)32136-1 31571602

[21] Hu G Han X Zhou H Liu Y. Public perception on healthcare services: evidence from social media platforms in China. Int J Environ Res Public Health. (2019) 16:1273. 10.3390/ijerph16071273 30974729 PMC6479867

[22] Mackey TK Liang BA. Combating healthcare corruption with improved global health governance. BMC Int Health Hum Rights. (2012) 12:23. 10.1186/1472-698X-12-23 23088820 PMC3519514

[23] Yip W Hsiao WC. China's health care reform: a tentative assessment. China Econ Rev. (2021) 30:309–20.31318101

[24] Huang Y Zhu Q. Game-theoretic frameworks for epidemic spreading and human decision-making: a review. Dyn Games Appl. (2022) 12:7–48. 10.1007/s13235-022-00428-0 35194521 PMC8853398

[25] Mostofi A Jain V Kumar S Mei Y Chandra C. A game theory data science-based mechanism for licensed pharmaceutical products concerning their deterioration: a case of a micro, small, and medium enterprise in Iran. Annals of Operations Res. (2023) 60:1–35. 10.1007/s10479-023-05360-z 37361076 PMC10204692

[26] Hausken K Ncube M. A game theoretic analysis of competition between vaccine and drug companies during disease contraction and recovery. Medical Deci Mak. (2022) 42:571–86. 10.1177/0272989X211053563 34738510 PMC9189729

[27] Bruzzone A Pusillo L. Perspective about Medicine Problems via Mathematical Game Theory: An Overview. IntechOpen (2020). 10.5772/intechopen.94488

[28] Zahedi F Farzaneh N. An evolutionary game theory–based security model in vehicular ad hoc networks. Int J Commun Syst. (2020) 33:e4290. 10.1002/dac.4290

[29] Feng L Lingheng L Han L. Evolutionary game and simulation analysis of competitive behavior of pharmaceutical enterprises in the context of centralized band purchasing. Econ Managem. (2023) 37:83–92.

[30] Shuhui S Qiang S. A four-way evolutionary game analysis of quality regulation of pharmaceutical research reports during major epidemics. Acad Manage J. (2020) 17:1391–401.

[31] Diamant M Baruch S Kassem E Muhsen K Samet D Leshno M. A game theoretic approach reveals that discretizing clinical information can reduce antibiotic misuse. Nat Commun. (2021) 12:1148. 10.1038/s41467-021-21088-5 33608511 PMC7895914

[32] Yeung H Makkapati S. Applying game theory models to inpatient medicine: opportunities to improve care. J Commu Hosp Inter Med Persp. (2023) 13:20. 10.55729/2000-9666.1135 36817294 PMC9924631

[33] Ding Y Li H Wang J. Game theory applications in the pharmaceutical industry: Business strategy optimization under market competition. J Bus Res. (2020) 113:204–15.

[34] Hausken K Ncube M. Game theoretic analysis of persons, the pharmaceutical industry, and donors in disease contraction and recovery. Humanit Soc Sci Commun. (2020) 7:1–17. 10.1057/s41599-020-00626-434297215

[35] Zhao K Yang ZF Zhuo Y Zhang S. Carbon emission reduction mechanism of the pharmaceutical supply chain: Quadrilateral evolutionary game models. Front Environ Sci. (2023) 11. 10.3389/fenvs.2023.1084343

[36] Hua M Tang H Lai IKW. Game theoretic analysis of pricing and cooperative advertising in a reverse supply chain for unwanted medications in households. Sustainability. (2017) 9:1902. 10.3390/su9101902

[37] Li X Ma Y. Financial reforms and regional investment conflicts in China: A game-theoretic analysis. Econ Planning. (1996) 29:117–30. 10.1007/BF00361418

[38] Tat R Heydari J Rabbani M. A mathematical model for pharmaceutical supply chain coordination: reselling medicines in an alternative market. J Clean Prod. (2020) 268:121897. 10.1016/j.jclepro.2020.121897

[39] Wen W Zhou P. Impacts of regional governmental incentives on the straw power industry in China: a game-theoretic analysis. J Clean Prod. (2018) 203:1095–105. 10.1016/j.jclepro.2018.08.149

[40] Zhu J. Do severe penalties deter corruption? A game-theoretic analysis of the Chinese case. China Rev. (2012) 12:1–32. Available at: https://cup.cuhk.edu.hk/chinesepress/journal/CR12.2/CR12.2_1-32.pdf

[41] Birkmire JC Lay JR Mcmahon MC. Keys to effective third-party process safety audits. J Hazard Mater. (2007) 142:574–81. 10.1016/j.jhazmat.2006.06.065 16887267

[42] Nikityuk V Karamavrova T Lebedynets V. The self-inspections (internal audits) process as a part of the pharmaceutical quality system: formation of a risk-based approach to internal audits planning. J Pharm Pharmacol. (2019) 7. 10.17265/2328-2150/2019.07.004

[43] Royce TJ Kircher S Conti RM. Pharmacy benefit manager reform: lessons from Ohio. JAMA. (2019) 322:299–300. 10.1001/jama.2019.7104 31219507 PMC7251257

[44] Mökander JFL. Operationalising AI governance through ethics-based auditing: an industry case study. AI and Ethics. (2023) 3:451–468. 10.1007/s43681-022-00171-7 35669570 PMC9152664

[45] Charoo NA Khan MA Rahman Z. Data integrity issues in pharmaceutical industry: Common observations, challenges and mitigations strategies. Int J Pharm. (2023) 63:1122503. 10.1016/j.ijpharm.2022.122503 36529357

